# From MEG to clinical EEG: evaluating a promising non-invasive estimator of defense-related muscle sympathetic nerve inhibition

**DOI:** 10.1038/s41598-023-36753-6

**Published:** 2023-06-12

**Authors:** John J. Eskelin, Linda C. Lundblad, B. Gunnar Wallin, Tomas Karlsson, Bushra Riaz, Daniel Lundqvist, Justin F. Schneiderman, Mikael Elam

**Affiliations:** 1 Institute of Neuroscience and Physiology, Department of Clinical Neuroscience, Sahlgrenska Academy at University of Gothenburg, 413 45 Gothenburg, Sweden; 2 Department of Clinical Neurophysiology, Sahlgrenska University Hospital, 413 45 Gothenburg, Sweden; 3 NatMEG, Department of Clinical Neuroscience, Karolinska Institutet, 171 77 Stockholm, Sweden

**Keywords:** Neurophysiology, Diagnostic markers

## Abstract

Sudden, unexpected stimuli can induce a transient inhibition of sympathetic vasoconstriction to skeletal muscle, indicating a link to defense reactions. This phenomenon is relatively stable within, but differs between, individuals. It correlates with blood pressure reactivity which is associated with cardiovascular risk. Inhibition of muscle sympathetic nerve activity (MSNA) is currently characterized through invasive microneurography in peripheral nerves. We recently reported that brain neural oscillatory power in the beta spectrum (beta rebound) recorded with magnetoencephalography (MEG) correlated closely with stimulus-induced MSNA inhibition. Aiming for a clinically more available surrogate variable reflecting MSNA inhibition, we investigated whether a similar approach with electroencephalography (EEG) can accurately gauge stimulus-induced beta rebound. We found that beta rebound shows similar tendencies to correlate with MSNA inhibition, but these EEG data lack the robustness of previous MEG results, although a correlation in the low beta band (13–20 Hz) to MSNA inhibition was found (p = 0.021). The predictive power is summarized in a receiver-operating-characteristics curve. The optimum threshold yielded sensitivity and false-positive rate of 0.74 and 0.33 respectively. A plausible confounder is myogenic noise. A more complicated experimental and/or analysis approach is required for differentiating MSNA-inhibitors from non-inhibitors based on EEG, as compared to MEG.

## Introduction

Transient inhibition of sympathetic nerve activity to blood vessels in human skeletal muscle tissue (muscle sympathetic nerve activity—MSNA) can be induced by introducing short-lasting and surprising stimuli^1^. This reaction has also been shown to correlate with blood pressure reactivity^2–4^, which is a risk factor for cardiovascular disease^5^. The central neural basis for this reaction has not been established, but several pieces of evidence suggest a link to cortical brain regions. For example, the inhibitory reaction is exaggerated in patients with phobia-induced vasovagal syncope, yet normal in comparable non-phobic fainters^6^. Furthermore, there is a negative correlation between the degree of sudden stimulus-induced MSNA inhibition and the amount of MSNA increase during longer lasting cognitive load^3^.

Recently it was shown that the inhibitory sympathetic response was in fact reflected in the cerebral cortex, namely the anterior cingulate cortex, a region implicated in autonomic regulation and the concepts of threat and defense reactions^7–10^. Both cortical thickness, as a measure of long-term plasticity, and a functional response in the form of neural oscillations in this region were correlated to inhibition^4^. Interestingly, a functional correlation was also discovered in the central sulcus region, i.e. sensorimotor cortex. Individuals with a high degree of MSNA-inhibition, as measured in a peripheral nerve, had increased event related synchronization in the beta band (13–25 Hz) in the contralateral sensorimotor cortex^4^. Such resynchronization is a well-known phenomenon found in the sensorimotor region following movement or stimulus presentation and is often referred to as *rebound*^11,12^. The physiological role of beta rebound is still a matter of debate and inquiry, but it has been suggested that beta oscillations reflect an increase in GABA signaling^13–15^, and that it serves to *maintain the status quo* of a cognitive set^12,16^.

The sympathetic inhibition elicited by transient stressors is believed to be an expression of the defense cascade^4^. The first phase of this can be called *arousal* as a state of heightened vigilance^17^. MSNA-inhibition can then be viewed as part of a primitive startup response before threat evaluation and a course of action has been determined. The inhibition of sympathetic activity to muscle makes sense as a way to decrease vascular resistance and increase blood flow in preparation for the later stages of the cascade such as fight-or-flight, but could also lead to fainting (play dead response) if sympathetic inhibition runs rampant^6^. The sympathetic nervous system is important in many areas of human pathology, and is likely to play a part in the increased risk for hypertension caused by an urban lifestyle which often includes small but recurrent threats to our physical, social, and economic wellbeing^18–20^. Our functional findings revealed a new and interesting link between traditionally dichotomized autonomic and voluntary branches of the nervous system.

The characterization of an individuals’ MSNA reactivity currently depends on microneurography, an invasive and cumbersome method of recording action potentials in vivo. The functional results in *Riaz *et al. were detected with magnetoencephalography (MEG). While being non-invasive, this is also a technique with limited availability, especially in clinical settings. Electroencephalography (EEG), on the other hand, is a widely utilized method in many clinics, most hospitals, and neuroscience research centers. Our principal aim with the present study was to explore whether sympathetic inhibition could be reliably characterized also with EEG using the same analysis approach. This could enable longitudinal high-powered clinical studies important for establishing a direct relationship to cardiovascular endpoints and risk for hypertension.

## Methods

### Participants

The study participants consisted of 49 young healthy males recruited from university notice boards; a summary of the analyzed participants’ characteristics is presented in Table 1. Exclusion criteria were set to any current medical condition or medication that might affect the nervous system or circulation. The study was approved by the regional ethics committee (Regionala Etikprövningsnämnden Göteborg, dnr 488-12, add T067-16, add T617-18) in accordance with the Declaration of Helsinki. All participants were informed in person and in writing of the nature and risks of the experiment and gave their written informed consent. All methods were performed in accordance with the relevant guidelines and regulations.

**Table 1:** Participants from both cohorts grouped by degree of MSNA-inhibition.

	Non-inhibitors (N = 19)				Inhibitors (N = 18)				All (N = 37)	
	Mean	StD	Min	Max	Mean	StD	Min	Max	Mean	StD
Age	26.3	5.8	21	41	29.2	8.0	19	45	27.7	7.0
BMI	25.2	3.6	19.8	34.1	23.4	4.0	18.0	34.4	24.3	3.8
SBP	119.7	10.5	97	136	116.0	8.8	103	135	117.9	9.8
MAP	81.9	6.7	67	92	83.2	8.3	67	97	82.6	7.5
DBP	63.8	7.2	50	77	68.0	10.6	43	93	65.8	9.1
HR	56.3	8.1	46	81	57.0	7.5	45	71	56.6	7.7
BI	41.5	13.6	11.7	66.9	46,0	14.7	21.7	72,4	43.7	14.1
BF	25.0	8.2	11.2	39.2	26.5	8.3	13.6	40.3	25.7	8.1

*StD* standard deviation of the mean, *Min* minimum value, *Max* maximum value, *BMI* body mass index, *SBP* resting systolic blood pressure, *MAP* resting mean arterial pressure, *DBP* resting diastolic blood pressure, *HR* resting heart rate, *BI* resting MSNA burst incidence (per 100 heart beats), *BF* resting MSNA burst frequency (per minute). There were no significant differences between groups.

### Experiment setup

The study material consisted of two cohorts that were examined with microneurography of sympathetic nerve activity and electroencephalography while experiencing somatosensory stimulation of the left index finger. The purpose of the microneurography was to characterize the level of sympathetic inhibition, induced by transient stimulation designed to be surprising and uncomfortable. Electroencephalography was performed to capture relevant induced cortical activity.

In the main cohort (29 out of 49 participants), EEG and MSNA recordings were done simultaneously. The stimulation protocol was identical to the one used in the MEG-sessions in *Riaz *et al. and consisted of 72 trials, with an inter-trial interval of 30, 45, 60 s in a pseudo-randomized order. Each trial contained three electrical stimulations (p1, p2, p3) locked to the R-wave of the preceding cardiac interval with a delay of 200 ms (cf below). Analysis required 1.5 s of uninterrupted data allowing one stimulus every other heartbeat (3 × 72 = 216 stims in total), and the multiple pulses allowed investigation of possible adaptation to repeated stimulation. Thus, the full analysis period encompassed six heartbeat intervals plus baseline (cf “Data analysis”). The stimulus strength of the transient pulses (0.2–0.8 ms) was tuned to a scale of 0–10 based on the subjective level of discomfort for the participant (0 no pain, 10 intolerable pain) aiming for a level of 7–8. The main cohort was supplemented by a second auxiliary cohort of 20 participants (from *Riaz *et al.) to obtain sufficient statistical power to enable reliable evaluation of a classification model of MSNA-inhibition. The main difference to the main cohort lay in the separate sessions for MSNA characterization and EEG-recordings. EEG data acquisition is described below; other details of this cohort and the experimental setup can be found in *Riaz *et al.

### Data acquisition

The following information concerns the main cohort. MSNA was acquired with microneurography from the left peroneal nerve using an insulated tungsten microelectrode with a tip of ~ 2 µm (1 or 5 MOhm; FHC, Bowdoin, USA), a subcutaneous reference and surface ground electrode. The signal was amplified by a factor of 40,000 and bandpass-filtered 0.7–2 kHz (Neuro Amp EX front-end and head stage; ADInstruments, Australia). Participants were comfortably resting in a seated semi-upright position. A 3-lead electrocardiogram (ECG) was monitored together with respiratory movements using a strain-gauge belt. For establishing the recording site, bursts of MSNA were identified by a set of predefined criteria^21^ and a 10 min period of resting activity was followed by instructions and initiation of the stimulation protocol, which lasted for about 1 h. MSNA-inhibition was calculated by comparing the average amplitude of post-stimulus bursts 0 and 1 to the baseline average amplitude measured from 8 cardiac intervals preceding stimulation ^22^. All electrical stimulations were locked and timed to 200 ms after the R-wave in the preceding heartbeat in order to optimize the induced inhibition^1^. Based on previous analyses of normal variability assessed from control/dummy trials, a significant degree of MSNA-inhibition has been determined as ≥ 30% reduction in average post-stimulus amplitude^22^. In accordance with previous studies, we found that approx. 50% of cases (main cohort: 9 inhibitors vs 11 non-inhibitors; auxiliary cohort: 10 vs 10) display a significant degree of inhibition^4,22^.

EEG was recorded with 19 channels according to the international 10–20 system, with a ground reference electrode in the left parieto-occipital region and one electrocardiogram lead and digitized at 256 Hz (Refa 32, Twente Medical Systems International, Netherlands). Electrodes were manually positioned with electrode gel (Elefix) after scrubbing the scalp with a peeling paste (abrasive gel, Everi). Participants were instructed to remain still and keep their eyes focused on a stationary cross to avoid ocular movement artefacts. The EEG in the auxiliary cohort was recorded with a MEG-compatible EEG cap (EasyCap, Brain Products GmbH) fitted with the same 19 channel array with the addition of Fpz and Oz for a total of 21 channels and digitized at 1000 Hz, during a joint session with MEG (Elekta Neuromag® TRIUX), during which an electro-oculogram and ECG was also obtained.

### Data analysis

Despite the fact that our two cohorts differed in terms of data acquisition and MSNA-characterization, they were merged in order to take advantage of the increased statistical power so the classification problem could be reliably addressed. All EEGs were re-referenced to a common average, bandpass filtered (Butterworth filter, 0.6–40 Hz) and resampled to 200 Hz. Analysis of the EEG focused on the Cz channel. The choice of electrode was based on the results of the previous study using MEG, which showed a strong correlation between MSNA inhibition and activations in the central sulcus area, and on its distance to cranial muscles, given that muscle artefacts may confound analysis of beta oscillations^23^. Eye-blinks and ECG artefacts were removed by employing the fast Independent Component Analysis algorithm found in MNE Python v0.20.8^24,25^. The number of components was determined by a fixed rank (i.e. no. of channels) and artefactual components were manually selected for removal after visual inspection. Typically, 4–6 components were rejected and filtered out.

The data was cropped into 1.5 s epochs locked to the time of the stimulus. Similarly, a control condition was created beginning at two heartbeats before the first stimulus and the data was baselined to the control condition, thus displaying change in frequency power from the baseline, expressed in dB. The 1.5 s epochs (baseline, p1, p2, p3) were visually inspected for artefacts. Artefacts were defined as being of technical origin, remaining eye-blinks, or a result of participant movement (e.g. electrode malfunctions, line noise, saccadic spike potentials, large amplitude deviations), or may have consisted of strong but transient myogenic noise in several channels (e.g. swallowing, coughing, speaking).

Because wakefulness dropped in some participants during longer sessions, all the EEGs were also screened for instances of sleep. Sleep-stage one^26^ is notoriously difficult to define and the time between stimulus trials was always 1 min or less making judgements of stage one in individual trials difficult. Therefore, reliable signs of sleep, i.e. (a) vertex sharp waves (b) K-complexes and (c) sleep-spindles, were marked in the raw data. As a safety measure, all stimulus trials within one minute before and one minute after such events were rejected in the EEG (and in concurrent nerve recordings in the main cohort). If the number of remaining trials in the EEG after cleaning both artefacts and sleep was less than 30, then the subject was excluded altogether. In total, 12 out of 49 participants were excluded from the statistical analysis: 2 due to low quality nerve registrations, either because the recording site was lost, or because of suspected involvement of sympathetic efferents to skin; 2 due to either a recurrent respiratory- or stimulation artefact in the EEG, which interfered with the frequency decomposition. The remainder were excluded due to insufficient remaining trials in the EEG. A total of 37 participants (cf Table 1) were included in the analysis and the average number of trial epochs rejected due to artefacts or sleep in EEG was 10.11 (SD 11.57), resulting in 5 participants with no trials excluded and the median number of included trials was 66 out of the maximum 72. Finally the EEG data was decomposed into power estimates in the frequency domain, in the range of 5–40 Hz with FieldTrip, revision 2020-06-07^27^. Sympathetic nerve recordings were also inspected visually for quality and an average of 2.73 (SD 7.38) trials were rejected due to artefacts and 2.22 (SD 6.14) due to sleep.

### Statistical analysis

Prior studies have shown that the upper beta band (20–30 Hz) and lower beta band (13–20 Hz) are susceptible to myogenic noise to different extents^23,28^. The frequency band was therefore divided (13–20 Hz: low beta; 20–30 Hz: high beta) and treated separately. Continuous time series of post-stimulus change in power in the low and high beta band, respectively, were computed for inhibitors and non-inhibitors. In order to test for statistically significant differences in the mean change in oscillatory power, we used a non-parametric cluster-based permutation test^29^. An independent two-sample *T* test (2-sided alpha = 0.05) in each time-point constituted the underlying cluster-forming test statistic. Clusters are then compared to a Monte-Carlo permutation (2-sided alpha = 0.05; 1000 permutations) for significance testing of the clusters based on the maximum size of the one-dimensional clusters. The non-parametric cluster-based permutation test was also used to test for the relationship between MSNA inhibition and the individual spectrograms of baseline corrected power. Spearman correlations were used as the underlying cluster-forming test statistic (2-sided alpha = 0.05; 1000 permutations). In this case, each cluster was evaluated for statistical significance against a Monte-Carlo permutation distribution based on the maximum size of the two-dimensional clusters.

We also employed a time–frequency window of interest. Inhibition was correlated to the average change in beta power from the baseline in a window encompassing 0.5–1.2 s post-stimulus and the beta frequency range (high or low) using Spearman’s correlation coefficient. In order to evaluate the prospects of using said window for non-invasive prediction of MSNA inhibition, we designed a simple binary classification model based on the significant correlations obtained wherein the two prediction categories were inhibitors and non-inhibitors. Non-inhibitors were defined as the positive category. The performance of this prediction model is summarized in a receiver-operating-characteristics (ROC)-curve of sensitivity vs 1 minus specificity and the area under the curve (AUC) and accuracy was computed.

## Results

**Figure 1:**
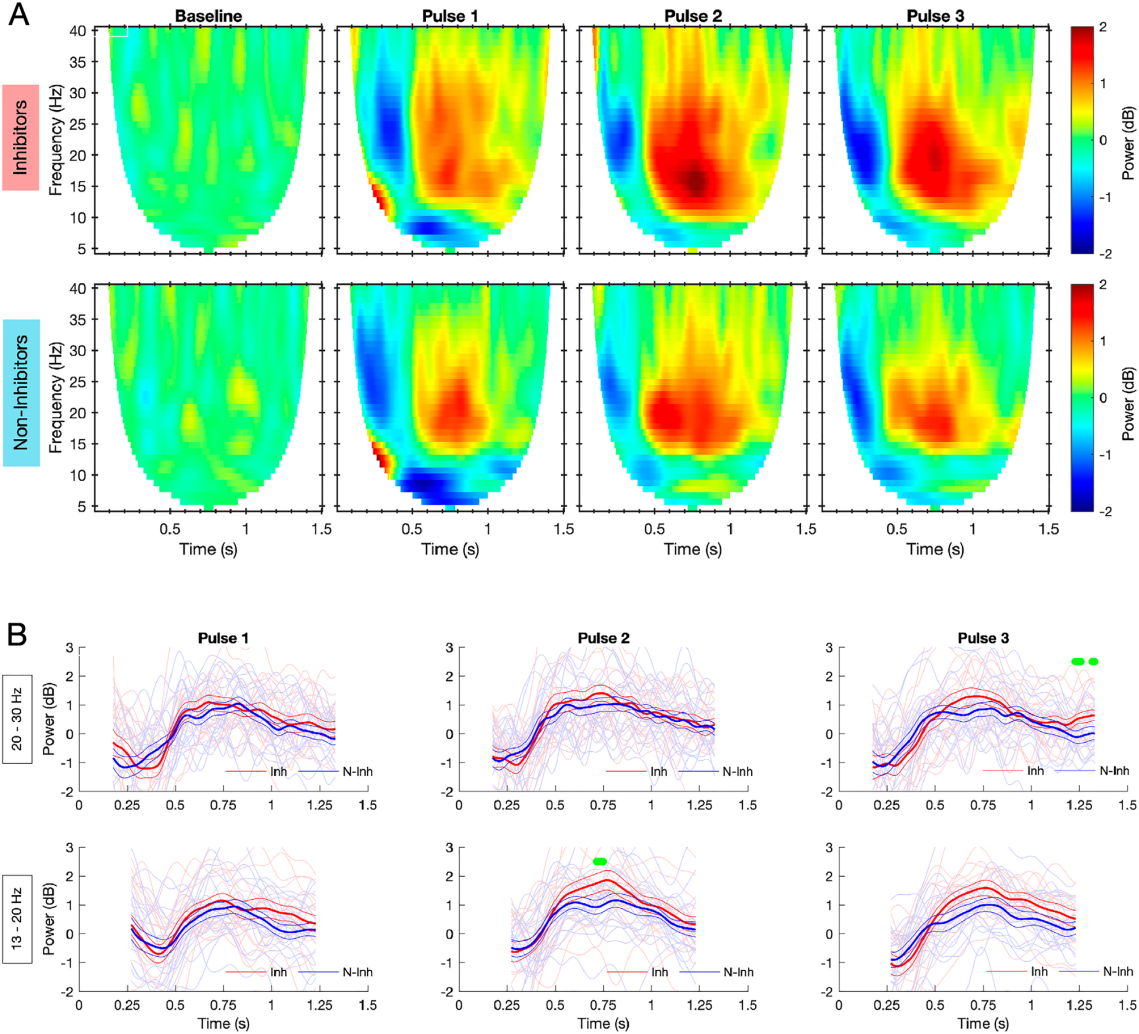
(**A**) Baseline corrected spectrograms of control (Baseline) period and post-stimulus (Pulses 1–3) neural oscillatory responses for all participants, divided into inhibitors (n = 18, top row) and non-inhibitors (n = 19). (**B**) Time-series evolution of average upper (20–30 Hz, top row) and lower (13–20 Hz, bottom row) beta oscillatory power for inhibitors (red) and non-inhibitors (blue). Saturated lines show group mean ± SEM, faded traces show individual means. The green colored bars on top show clusters (before correction for multiple comparisons) of significant differences between groups: multiple independent 2-sample *T* tests, p < 0.05.

Responses to stimulation are shown as analyzed in the frequency domain, revealing a decrease in oscillatory power, indicative of desynchronization of neural oscillations, followed by resynchronization and subsequent overshoot in power predominantly in the beta frequency range 13–30 Hz (Fig. 1A) i.e. beta rebound. The cluster-based permutation test returned no significant clusters correlated with MSNA-inhibition when applied to the baseline corrected spectrograms. The time-series of upper and lower beta bands (Fig. 1B) suggest a modest tendency for increased beta rebound in inhibitors compared to non-inhibitors. However, the clusters of primarily significant *T* tests on the time-series data did not survive correction for multiple comparisons.

**Figure 2:**
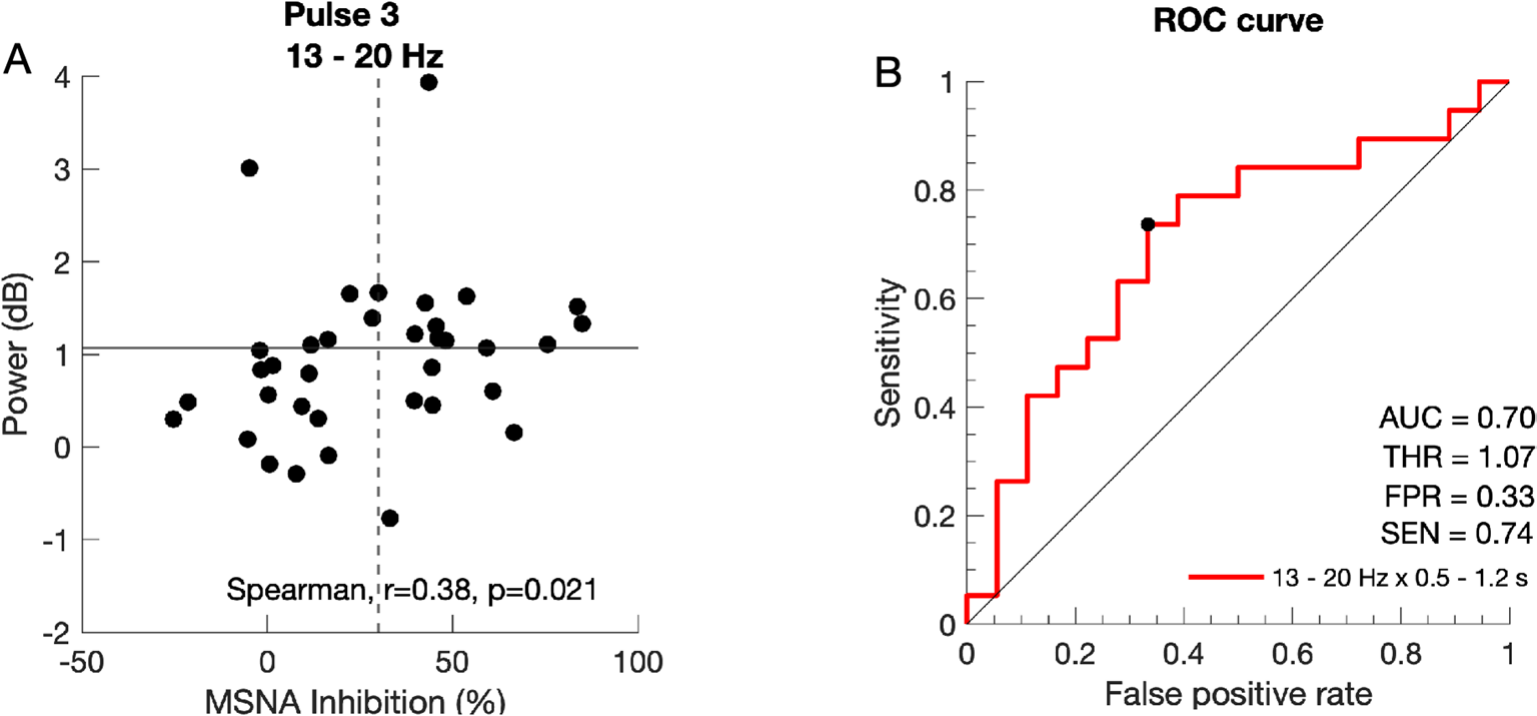
(**A**) Spearman correlation between individual MSNA-inhibition and average oscillatory power within the lower beta band (13–20 Hz) and 0.5–1.2 s post stimulus in pulse 3 (r = 0.38, p = 0.021); dashed vertical line shows cutoff between inhibitors and non-inhibitors; horizontal line shows optimal threshold for binary classifier. (**B**) Receiver-operating-characteristic (ROC) performance curve of the binary classifier. Each step represents a different meaningful threshold and the threshold with the optimum trade-off between sensitivity and FPR is marked. *THR* optimum threshold (dB), *AUC* area under the curve, *SEN* sensitivity, *FPR* false positive rate.

A significant correlation between the individual level of MSNA inhibition and average lower beta oscillatory power (13–20 Hz, 0.5–1.2 s) was found in p3 (Fig. 2A, Spearman, r = 0.38; p = 0.021). To attempt to answer the question of whether this surrogate measure is a reliable substitute for invasive measurements using microneurography, a binary classifier was constructed based on the correlation in p3. The performance of the binary classification is summarized in a receiver-operating-characteristic curve in Fig. 2B. The performance curve shows that the threshold for optimal trade-off between sensitivity and specificity achieves a sensitivity of 0.74 at the cost of a 0.33 false-positive rate when non-inhibitors are defined as cases (Accuracy = 0.70; AUC = 0.70). This threshold corresponded to a change in power of 1.07 dB in the lower beta band 13–20 Hz.

## Discussion

Here we explored the feasibility of using EEG as a surrogate method for characterization of defense-related sympathetic inhibition. While a correlation between the stimulus-induced beta rebound response recorded with EEG and the microneurographically determined MSNA inhibition was found, this relationship was weak using EEG compared to our previous study using MEG. Thus, using a similar analysis approach, the group differences and the results of a simple binary classifier based on previous MEG findings were not convincing. Our interpretation is that EEG data in the beta-activity range is compromised by cranial muscle activity, highlighting the benefit of MEG in stress-related studies of higher frequency cortical oscillations.

First it should be noted that the time–frequency window of interest used herein was an approximation and interpretation of the likely extent of an effect indicated by the clusters found with MEG. The reasons for this approach are that (1) it is not possible to exactly determine the extent of an effect using the cluster-based permutation test as it is dependent on arbitrary factors like signal-to-noise ratio and mainly serves to test the null hypothesis. And we now use a different modality so (2) it follows that a soft interpretation avoids overfitting the model to the data it was first based on, which would otherwise risk decreased external validity and sensitivity. Here we attempted to avoid the well-known problem of contamination from muscle activity by separating the beta band into higher and lower frequencies; typically, 20 Hz defines this boundary^30^. Indeed, the lower beta band did reveal a significant relationship with MSNA-inhibition, whereas the higher beta band did not.

The cluster-based permutation test provides an unbiased data-driven way to detect focal effects without the need for a predefined window of interest and should thus increase sensitivity. However, testing for significant clusters in the baseline corrected power spectrograms, as done in *Riaz *et al. did not yield any significant results post correction (see Supplementary material: Fig. S1, for time–frequency diagrams showing the raw test statistics). The same principle can be applied to one-dimensional data as in the time series of beta power, but likewise no robust statistical differences were detected in the average beta power over time.

The binary classification derived from the significant correlation between average power of the low beta time–frequency window of interest and MSNA-inhibition achieved an AUC score of 0.70 (> 0.50 denotes positive skill). The mathematically optimal threshold, based on a trade-off between sensitivity and specificity, also gives an accuracy of 0.70, but is not necessarily the best one for any given purpose. We hypothesize that the non-inhibitors would be at greater cardiovascular risk due to their tendency for blood pressure increases, and so it is more important to focus on this group in a clinical setting. But inspection of the ROC-curve shows that even a threshold that stays below a FPR of 20% only reaches about 45% sensitivity. As it currently stands, the performance curve suggests that the prospects for models of EEG-based sensorimotor beta rebound may be limited, and this particular model is far from being useful in smaller studies like this one. The model would thus likely require very large sample sizes for clinical research, in order to connect MSNA-inhibition to cardiovascular end-points.

It is likely that myogenic noise interfered with signals of neural origin, especially in higher frequencies. It has been shown that even in a relaxed state, residual muscle activity exerts a significant influence on frequencies mainly from ~ 20 Hz and above, but can be visible around the vertex on frequencies as low as 12 or 8 Hz^23,28^. Additionally, single motor unit potentials originating from the temporalis muscle have been shown to travel across the entire scalp, reaching the contralateral side of an EEG recording^31^, and the temporalis muscle displays a peak at about 20 Hz, which is in the center of the beta band^28^. Yilmaz et al. further argue that low-pass filtering should be set as high as 1500 Hz, far from the common 70 Hz cut-off, in order to avoid single motor units being smoothened into “beta-like” activity. Indeed, due to the phenomenon of volume conduction, EEG is more susceptible than MEG to activity generated by muscle tissue^30^, which serves to explain the discrepancy compared to *Riaz *et al. Volume conduction also results in lower spatial specificity for neural sources. Thus, the oscillatory response may mix with other nearby sources including more frontal and parietal areas of the cortex, which is not the case for source-localized MEG. The source localization in MEG makes powerful use of spatial filtering and information from individual MRIs. While source localization can, in principle, be done with EEG, it is not as precise, relies on more assumptions (e.g. regarding tissue conductivities) and is preferably done with a larger number of channels^32,33^. Here we wanted to work towards an easily accessible instrument that could be incorporated into clinical routine, hence a common 19 channel montage. The streaky appearance of short intermittent, highly variable shifts in power found in the upper beta and gamma range seem consistent with sporadic muscle potentials. Furthermore, during data analysis and inspection of individual spectrograms of the response to stimulation, a notable interindividual variability was observed for peak responses. Thus, targeting of the lower beta spectrum may increase specificity in EEG, but will possibly also reduce sensitivity.

It is not beyond reason that the very long and demanding sessions of simultaneous recordings may have contributed with undesirable variability due to diminished wakefulness. Because cortical activity is influenced by wakefulness and it has previously been shown that K-complexes in sleep stage 2 can give rise to MSNA bursts^34,35^, we searched for and excluded trials that indicated light sleep. The conventional clinical characterization of sleep stage 1 is based on judging levels of alpha (8–12 Hz) activity during 20–30 s periods^26^. It is notoriously difficult to reliably classify and more importantly, participants were instructed to keep their eyes open while alpha is normally mainly visible with eyes closed. We would also have required more time between trials for this approach to work well. Instead, we opted for more reliable indices combined with a safety margin around each marked trial (cf Methods). Seven of the included participants had some indication of sleep and the number of rejected trials, including margins, ranged from 3 to 32 with a median of 10 (the most extreme case was of good quality during the first half while the second half was dropped) and the average number of rejected trials due to sleep was 2.2 (SD 6.1), when measured across all participants.

### Limitations

While this study is limited by the exclusion of female participants, it is important to recognize the different hemodynamic and sympathetic activity profiles of men and women^36^. We have chosen to focus first on men because they are at higher risk earlier in life. However, several female cohorts have been examined with the same protocol in our lab and results are being prepared for publication.

The fact that a slightly different stimulus protocol was used during nerve recording in our auxiliary cohort may be seen as a limitation, but since we calculate MSNA-inhibition based on the first stimulus in stimulus trains, we assume this issue has little or no impact. The stimulus regime for eliciting cortical responses was identical in both cohorts, and the circumstances during recording in a MEG-chamber and our microneurography lab are similar. The main difference was the longer duration of the simultaneous registrations used in our main cohort, which turned out to be taxing for some individuals as it resulted in diminished wakefulness.

Our limited EEG analysis exploration could be regarded as a limitation. However, the primary aim of this work was to explore the possibility of transferring the MEG-derived classification model for non-invasively characterizing sympathetic response profiles to the more clinically-accessible EEG. The analysis we performed herein was thus purposefully limited in scope to match that which we utilized successfully on our previous MEG data. It is also worth mention that we have attempted a variety of more complicated AI-based methods (including data augmentation, long short term memory, convolutional, and feedforward neural networks) to classify our previous MEG data related to MSNA, but to no avail. Because the EEG data obtained herein showed weaker correlations to MSNA than we have previously reported with MEG data, we elected not to pursue the development of AI-based classification methods on this EEG data.

## Conclusions

The tendency for a relationship between increased spectral power in the beta band and significant MSNA-inhibition can be observed in EEG. While the analysis performed herein is not exhaustive, it was based on promising MEG-results. Because EEG-detected beta oscillations are relatively more vulnerable to myogenic noise (as compared to MEG), a more complicated experimental and/or analysis approach is therefore required if EEG-based measurements are to be utilized as a reliable and robust non-invasive surrogate method reflecting defense-related sympathetic inhibition in clinical routine. MEG thus remains as a more straightforward method for further research in the field due to its good spatial precision and excellent temporal resolution. Although the availability of MEG is somewhat limited at present, there are projects racing towards engineering a more accessible next generation system^37,38^. Our long-term aim with studying these defense-related MSNA responses is, for us and others, to explore the relationship between sympathetic defense reactions to external stimuli and circulatory homeostasis on a grander scale. The fact that these sympathetic responses have been shown to not be genetically determined^22^ suggests that there is potential to influence them in a homeostatically favorable direction.

## Supplementary Information

Supplementary Figure S1.

## Data Availability

The datasets generated during and/or analyzed during the current study are available from the corresponding author on reasonable request.
