# Supplementary material for: From MEG to clinical EEG: evaluating a promising non-invasive estimator of defense-related muscle sympathetic nerve inhibition
Source: Sci Rep. 2023 Jun 12;13:9507. doi: 10.1038/s41598-023-36753-6 (PMC10261137; doi:10.1038/s41598-023-36753-6)
Supplement: Supplementary file 1 — Supplementary Figure S1. [file 41598_2023_36753_MOESM1_ESM.docx]

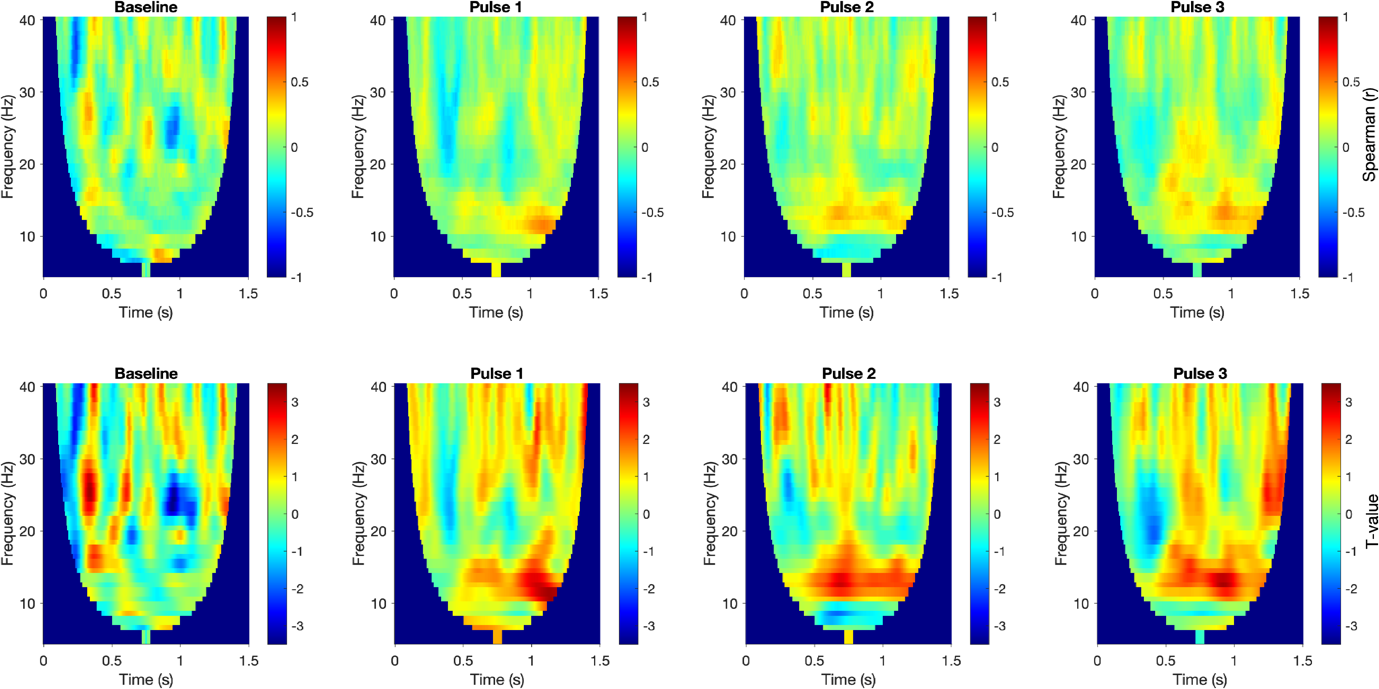


Figure S1. Raw statistics for r-values and T-values when comparing oscillatory power changes to MSNA-inhibition in each point of the time-frequency response diagram. Top: Non-parametric Spearman correlation coefficients. Bottom: T-values from independent samples T-tests comparing inhibitors and non-inhibitors. There is a tendency towards larger clusters in time-frequency space in the low beta range. However, no clusters are designated as significant post correction using a non-parametric cluster-based permutation test (see methods section in paper).
